# Motor-to-Limbic Design of Direct Synaptic Communication between Dopamine Neurons in the Midbrain

**DOI:** 10.1523/JNEUROSCI.2126-25.2026

**Published:** 2026-04-28

**Authors:** Niklas Hammer-Bahador, Guilian Tian, Beatrice Fischer, Strahinja Stojanovic, Kevin Beier, Jochen Roeper

**Affiliations:** ^1^Institute of Neurophysiology, Goethe University Frankfurt, Frankfurt am Main 60590, Germany; ^2^Department of Physiology and Biophysics, University of California, Irvine, California 92617

**Keywords:** antipsychotics, autoreceptor, basal ganglia, dopamine, substantia nigra

## Abstract

Midbrain dopamine (DA) neurons are diverse with distinct subpopulations being essential for key functions of the brain: nigrostriatal DA neurons for voluntary movement and mesolimbic DA neurons for learning from reward prediction errors. In addition to being primarily associated with distinct senso-motor or limbic cortical-striatal circuits, DA subpopulations also directly communicate with each other via local DA release in the midbrain. While the inhibitory synaptic nature of this dopamine-to-dopamine signaling has been well established, the pre- and postsynaptic identity and logic of connectivity among DA subpopulations are still unresolved. To fill this gap, we combined retrograde tracing with projection-specific optogenetic stimulation of DA neurons and patch-clamp recordings in vitro in the adult mouse of either sex. We functionally identified a unidirectional, motor-to-limbic design of the DA synapse in the midbrain. This motor-to-limbic negative feedback connection in the midbrain was independently confirmed by monosynaptic rabies tracing of projection-defined DA subpopulations. This DA synapse might complement the limbic-to-motor striato-nigro-striatal feedforward architecture of the basal ganglia.

## Significance Statement

We identified the pre- and postsynaptic partners of the dopamine-to-dopamine synapse in the midbrain, independently by functional in vitro patch-clamp recordings and monosynaptic rabies tracing of identified dopamine subpopulations. This DA synapse is surprisingly circuit specific with presynaptic DA neurons projecting to the dorsal striatum and postsynaptic DA neurons projecting to the lateral shell of the nucleus accumbens. Thus, this DA synapse establishes a unidirectional, direct communication between the nigrostriatal and the mesolimbic dopamine systems.

## Introduction

Midbrain dopamine (DA) neurons located in the ventral tegmental area (VTA) and the substantia nigra (SN) play a crucial role in different brain functions, such as voluntary movement, encoding of reward prediction errors, aversion, and novelty (Schultz et al., 1997; Menegas et al., 2017; da Silva et al., 2018; de Jong et al., 2019). Furthermore, impairments in the DA system play a central role in various diseases such as schizophrenia, depression, substance use disorder, or Parkinson disease (Grace, 2016; Surmeier et al., 2017; Volkow et al., 2017).

Based on their axonal projection sites, DA neurons are part of different circuits and encode various information (Morales and Margolis, 2017; Coddington and Dudman, 2019). DA neurons located in the SN project to the dorsomedial striatum (DMS) and dorsolateral striatum (DLS), DA neurons located in the VTA project to the ventral striatum, e.g., the lateral shell of nucleus accumbens (lNAcc), or the medial shell of nucleus accumbens (mNAcc; Lammel et al., 2008; Farassat et al., 2019). While DA neurons projecting to the DLS play a central role in fast dynamics in motor-related behavior, lNAcc-projecting DA neurons primarily encode reward prediction errors. In addition, mNAcc-projecting DA neurons signal in response to aversive stimuli (de Jong et al., 2019; Markowitz et al., 2023; de Jong et al., 2024). Furthermore, these different subpopulations possess molecular differences (Poulin et al., 2018) and receive different synaptic inputs from cortico-striatal, hypothalamic, and brainstem sources (Watabe-Uchida et al., 2012; Beier et al., 2015; Lerner et al., 2015).

In addition to distal axonal DA release in projection targets, DA neurons also release DA in the midbrain (Geffen et al., 1976), which act upon somato-dendritic type 2 dopamine receptors (D2R; Ford, 2014). Activation of D2R leads to membrane hyperpolarization by activation of GIRK channels, therefore inhibiting pacemaker firing of DA neurons (Lacey et al., 1987; McCall et al., 2017; Gantz et al., 2018).

Both extrasynaptic and synaptic dopamine release sites as well as D2R have been described in the midbrain (Sesack et al., 1994). In the VTA, axo-dendritic and dendro-dendritic dopamine-to-dopamine synapses have been reported using electron microscopy (Bayer and Pickel, 1990). In line with these anatomical findings and differentiated by their calcium dependence, DA release in the VTA shows both, putative axonal and a somato-dendritic components (Chen et al., 2011). Moreover, DA synaptic input onto VTA DA neurons has been detected using rabies virus monosynaptic input tracing (Beier, 2022), though it is unclear whether rabies can discriminate between axo-dendritic and dendro-dendritic synapses.

In the SN only dendro-dendritic synapses have been described using electron microscopy (Wilson et al., 1977; Groves and Linder, 1983; Bayer and Pickel, 1990). In addition, DA release in the SN shows a calcium dependence consistent with somato-dendritic component (Chen et al., 2011). Furthermore, activity-dependent autoinhibition has been described for DA neurons located in the SN (Hikima et al., 2021). Activation of these dopamine-to-dopamine synapses in the VTA and the SN results in a D2R-mediated evoked inhibitory postsynaptic currents (eIPSCs) or spontaneous IPSPs (Beckstead et al., 2004; Ford et al., 2009; Gantz et al., 2013).

However, the pre- and the postsynaptic identities for this DA-to-DA synapse has remained unresolved.

Here, we used retrograde tracing combined with electrical stimulation, as well as projection-specific optogenetic activation of defined presynaptic DA subpopulations to characterize the DA-to-DA synapse and contrast it with DA volume transmission. In addition, we employed monosynaptic rabies-mediated identification of presynaptic DA inputs onto projection-identified DA neurons to demonstrate the anatomical patterns of connectivity between midbrain DA neurons.

## Materials and Methods

### Animals

C57BL/6N mice (Charles River Laboratories), male, aged 8–12 weeks were used for recordings of eIPSCs. For experiments involving floxed viral constructs, expression was restricted to DA neurons using DAT-Cre mice (Zhuang et al., 2005; Slc6a3^tm1(cre)Xz^/J; Jackson Laboratory, stock no.: 020080 backcrossed to C57BL/6N mice for six generations) of both sexes aged 13–16 weeks. Mice were group-housed; when possible, were maintained on a 12 h light/dark cycle; and had a*d libitum* access to food and water. All experimental procedures were performed during the light phase of the cycle. All procedures were approved by the Regierungspräsidium Darmstadt (FU/1257; FU/2026). All procedures involved RABV input mapping were approved by UCI's Institutional Animal Care and Use Committee (AUP-24-117) and Institutional Biosafety Committee (BUA-R261).

### Chemicals

All drugs and chemicals were obtained from Merck, Tocris Bioscience, Alomone Labs, Cayman Chemical, or BioTrend. Drugs were dissolved in H_2_O, 1 M NaOH, or DMSO and stored at −20°C until use.

### Virus

pAAV-EF1a-double-floxed-hChR2(H134R)-EYFP-WPRE-HGHpA was a gift from Karl Deisseroth (Addgene viral prep #20298-AAV9; http://n2t.net/addgene:20298; RRID: Addgene_20298). AAV pEF1a-DIO-FLPo-WPRE-hGHpA was a gift from Li Zhang (Addgene viral prep #87306-AAVrg; http://n2t.net/addgene:87306; RRID:Addgene_87306).

AAV-FLEx^FRT^-TC^66T^-mCherry, AAV-FLEx^FRT^-RABV-G, and RABVΔG were made in the Beier Lab. Viral preparations were stored at −80°C until use.

### Stereotaxic surgeries

Stereotaxic injections were performed as described previously (Hammer et al., 2024). In brief, animals were anesthetized with isoflurane (AbbVie; 0.8–1.4% in 0.35 L/min O_2_), and local anesthesia was provided using lidocaine/prilocaine gel (EMLA cream, AstraZeneca). Depth of anesthesia, body temperature, and respiratory rate were continuously monitored throughout the procedure.

Specific brain regions were targeted relative to bregma as follows: dorsal striatum (DS: anterior +0.74 mm, lateral ±1.6 mm, ventral −2.6 mm), dorsomedial striatum (DMS: anterior +0.74 mm, lateral ±1.2 mm, ventral −2.6 mm), dorsolateral striatum (DLS: anterior +0.74 mm, lateral ±2.2 mm, ventral −2.6 mm), lateral shell of the nucleus accumbens (lNAcc: anterior +0.86 mm, lateral ±1.75 mm, ventral −4.5 mm), tail of the striatum (TS: anterior −1.0 mm, lateral ±2.8 mm, ventral −2.7 mm), and medial shell of the nucleus accumbens (mNAcc: anterior +1.54 mm, lateral ±0.45 mm, ventral −4.1 mm). Coordinates were corrected as reported previously (Lammel et al., 2008). To enable optogenetic activation of defined DA subpopulations, 130 nl of AAV9-EF1a-double-floxed-hChR2(H134R)-EYFP (titer: 1.8 × 10^13^ vector genomes/ml) were injected unilaterally. After >28 d to allow sufficient viral expression, animals underwent a second surgery in which red RB were injected.

For retrograde tracing, 100 nl of Red Beads (Lumafluor), diluted 1:30 in ACSF (Harvard Apparatus), or 100 nl of undiluted Green Beads (Lumafluor) were injected into the target area using a 1 µl Hamilton syringe (Hamilton). Animals were then allowed to recover for three days to permit sufficient retrograde axonal transport of the beads.

### Slice preparation

Slice preparation was performed as described previously (Hammer et al., 2024). Animals were killed by intraperitoneal injection of a lethal dose of ketamine (1 mg/g; Bela-Pharm) and medetomidine (10 µg/g; Zoetis). Subsequently, mice were transcardially perfused with ice-cold artificial cerebrospinal fluid (ACSF) containing the following (in mM): 125 NaCl, 2.5 KCl, 6 MgCl_2_, 0.1 CaCl_2_, 25 NaHCO_3_, 1.25 NaH_2_PO_4_, 50 sucrose, 2.5 glucose, and 3 kynurenic acid, continuously bubbled with carbogen (95% O_2_/5% CO_2_). Brains were rapidly removed, and 250 µm coronal slices containing the midbrain were cut using a vibratome (VT1200S, Leica).

Slices were allowed to recover for 1 h at 37°C in ACSF containing (in mM): 125 NaCl, 3.5 KCl, 1.2 MgCl_2_, 1.2 CaCl_2_, 25 NaHCO_3_, and 1.25 NaH_2_PO_4_, continuously bubbled with carbogen (95% O_2_/5% CO_2_). After recovery, slices were maintained at room temperature until use for electrophysiological recordings.

### In vitro electrophysiology

Brain slices were placed in a recording chamber continuously superfused with ACSF containing the following (in mM): 125 NaCl, 3.5 KCl, 1.2 MgCl_2_, 2.4 CaCl_2_, 25 NaHCO_3_, and 1.25 NaH_2_PO_4_, bubbled with carbogen (95% O_2_/5% CO_2_) at a rate of 2–4 ml/min. Temperature was controlled using a Temperature Controller (Temperature Controller VI, Luigs & Neumann), set to 37°C, resulting in a bath temperature of 30–31°C.

**Figure 1.**
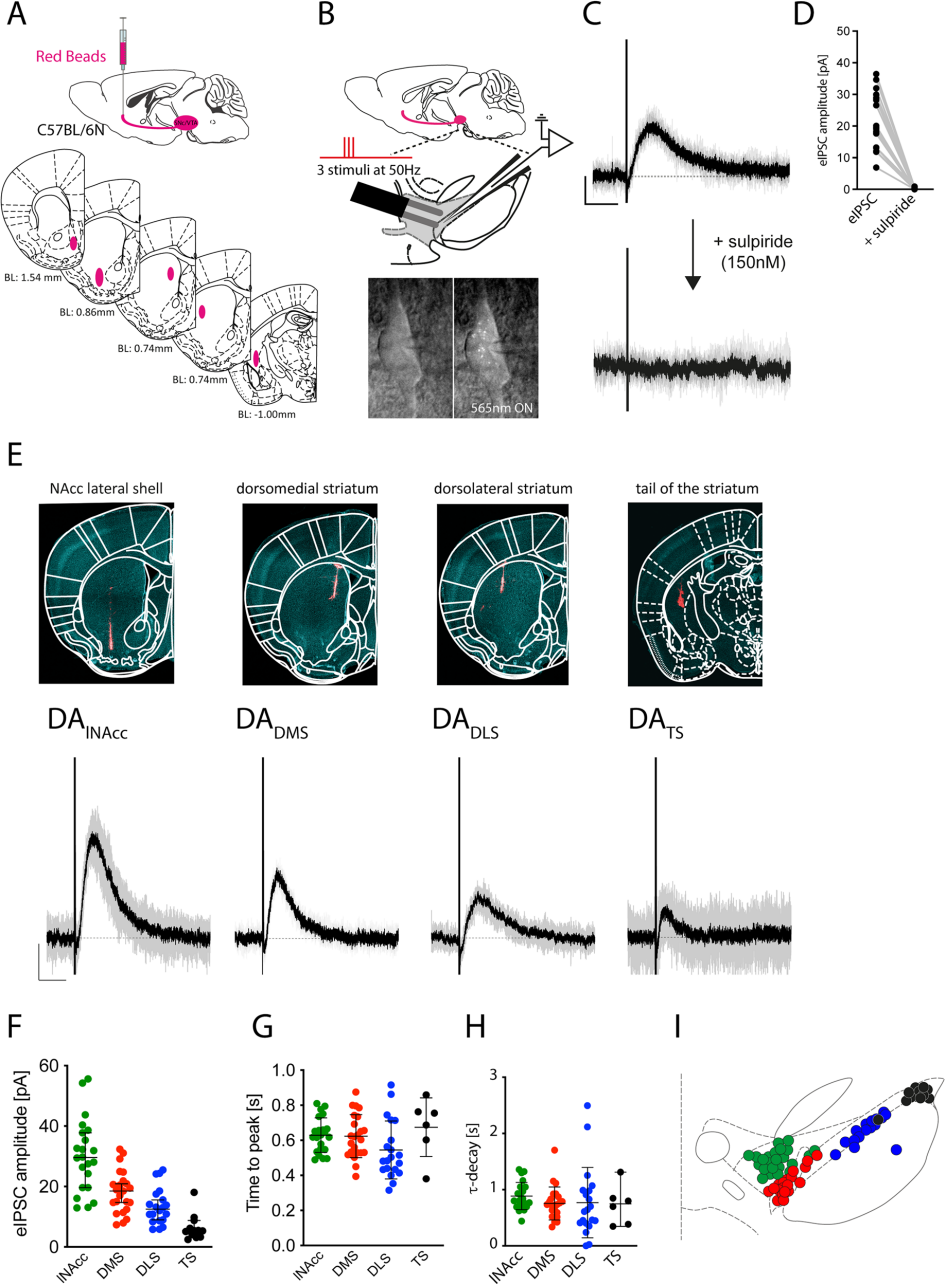
Medial-to-lateral gradient of D2R-eIPSCs is axonal projection specific. ***A***, Experimental design. C57BL/6N mice received 100 nl injections of Red Beads (RB) into defined striatal regions for retrograde labeling of midbrain dopamine (DA) neurons according to their axonal projection site. ***B***, Acute coronal midbrain slices containing the substantia nigra pars compacta (SN) and ventral tegmental area (VTA) were prepared. RB-labeled DA neurons were visualized under 562 nm excitation and recorded in whole-cell voltage-clamp configuration, while surrounding cells were stimulated using a bipolar electrode (three pulses, 50 Hz). ***C***, Representative voltage-clamp recording from a RB-positive lNAcc-projecting DA neuron at −55 mV. Electrically evoked inhibitory postsynaptic currents (eIPSCs) were pharmacologically isolated using dʟ-AP5, CNQX, SR95531, CGP55845, mecamylamine, CPCCOEt, MPEP, and prazosin. Bath application of 150 nM sulpiride abolished the outward current, confirming D2R-mediated signaling. Calibration: 1 s, 10 pA. ***D***, eIPSC amplitudes before and after bath application of sulpiride (control: 19.3 pA [17.1–25.8]; sulpiride: 0.0 pA [0.0–0.3]; *n* = 18 cells, *N* = 8 mice; two-tailed Wilcoxon matched-pairs signed rank test, *W* = −171, *p* < 0.0001). ***E***, RB injection sites in striatal subregions (blue, DAPI; red, RB, top). Representative eIPSC traces from RB-positive DA neurons projecting to the lateral shell of the nucleus accumbens (DA_lNAcc_), dorsomedial striatum (DA_DMS_), dorsolateral striatum (DA_DLS_), and tail of the striatum (DA_TS_; bottom). Calibration: 10 pA, 1 s. ***F***, Projection-specific gradient of D2R-eIPSC amplitudes. DA_lNAcc_: 29.6 pA [24.8–35.4], *n* = 22 cells, *N* = 8 mice; DA_DMS_: 18.5 pA [15.2–21.3], *n* = 22 cells, *N* = 7 mice; DA_DLS_: 12.5 pA [10.6–16.2], *n* = 20 cells, *N* = 7 mice; DA_TS_: 5.4 pA [3.7–9.1], *n* = 12 cells, *N* = 3 mice; one-way ANOVA, *F*_(3,73)_ = 25.74, *p* < 0.0001. ***G***, Time-to-peak values did not differ significantly. DA_lNAcc_: 0.629 ± 0.021 s, *n* = 22 cells, *N* = 8 mice; DA_DMS_: 0.624 ± 0.026 s, *n* = 23 cells, *N* = 7 mice; DA_DLS_: 0.545 ± 0.037 s, *n* = 20 cells, *N* = 7 mice; DA_TS_: 0.675 ± 0.068 s, *n* = 6 cells, *N* = 3 mice; one-way ANOVA, *F*_(3,69)_ = 1.689, *p* = 0.1774. ***H***, No significant differences in decay constants (τ-decay). DA_lNAcc_: 0.821 s [0.778–0.993], *n* = 22 cells, *N* = 8 mice; DA_DMS_: 0.735 s [0.628–0.882], *n* = 23 cells, *N* = 7 mice; DA_DLS_: 0.628 s [0.478–1.062], *n* = 20 cells, *N* = 7 mice; DA_TS_: 0.749 s [0.361–1.102], *n* = 6 cells, *N* = 3 mice; one-way ANOVA, *F*_(3,67)_ = 0.5061, *p* = 0.6794. ***I***, Post hoc localization of recorded DA neurons revealed a projection-specific topography (green, DA_lNAcc_; red, DA_DMS_; blue, DA_DLS_; black, DA_TS_). All data are presented as mean ± SEM or median (95% CI).

DA neurons were visualized using a light microscope (Axioskop 2FS Plus, Zeiss) equipped with an infrared light source (SOLIS-850C, Thorlabs) to prevent cross-activation of ChR2 and a CMOS camera (CS505MUP1, Thorlabs). RB were excited with a LED lamp (SOLIS-1C, Thorlabs) using a 562/40 nm bandpass filter. After 5–15 min RB-positive neurons were patched with borosilicate glass pipettes (2–4 MΩ, GC150TF, Harvard Apparatus) pulled with a horizontal pipette puller (DMZ Universal Electrode Puller, Zeitz) in whole-cell configuration. Pipettes were filled with an internal solution containing the following (in mM): 135 K-gluconate, 5 KCl, 10 HEPES, 0.1 EGTA, 5 MgCl_2_, 0.075 CaCl_2_, 5 Na_2_ATP, 1 LiGTP, and 0.1% neurobiotin; pH adjusted to 7.35 with KOH, 290–300 mOsmol. To block Kv channels in the recorded neuron, 5 mM of 4-AP was added to the internal solution. DA neurons were recorded using an EPC-10 USB Double Amplifier (HEKA Elektronik) at a sampling rate of 20 kHz. Signals were filtered with a 5 kHz low-pass Bessel filter. Neurons were voltage clamped at a holding potential of −55 mV. Series resistances were <15 MΩ and were electrically compensated by 75%. Midbrain slices were stimulated with a bipolar electrode (PBSA02575, FHC) with a tip separation of 250 µm, positioned 125 µm medial or lateral to the recorded cell. Three stimuli (50 Hz, applied every 60 s, 2–10 mA) were delivered through a stimulus isolator (A360, WPI) to elicit eIPSCs. No correlation between stimulation intensity and peak amplitude was observed [linear regression: *y* = −0.032*x* + 8.73; *R*^2^ = 0.023, *p* = 0.151 (slope)]. Furthermore, stimulation intensity did not differ between different projection sites (Kruskal–Wallis's test, *H*_(4)_ = 6.93, *p* = 0.1399). Only one neuron per slice was patched. To pharmacologically isolate DA transmission, the slice was superfused with a blocking cocktail containing dʟ-AP5 [10 µM (Hammer et al., 2024), BioTrend], gabazine [SR95531, 4 µM (Hammer et al., 2024), Tocris], CNQX [20 µM (Liss et al., 2005), Tocris], MPEP (200 nM, Tocris), CPCCOEt [50 µM (Ledonne and Mercuri, 2018), Tocris], mecamylamine [20 µM (Tsuneki et al., 2000), Tocris], prazosin [100 nM (Beckstead et al., 2004), Tocris], and CGP55845 [50 nM (Stojanovic et al., 2025), Tocris]. Bath application of sulpiride [150 nM (Beckstead et al., 2004), Tocris] abolished the eIPSC (Fig. 1).

To block DAT, potassium, or calcium channels, specific blockers were applied in combination with the above cocktail: AmmTx3 [1 µM (Stojanovic et al., 2025), Alomone Labs], apamin [300 nM (Stojanovic et al., 2025), Tocris], cadmium chloride [300 µM (Beckstead et al., 2004), Sigma-Aldrich], dendrotoxin-K [100 nM (Gittelman and Tempel, 2006), Alomone Labs], GBR 12783 [200 nM (Ferraro et al., 2012), Tocris], isradipine [300 nM, preincubated >10 min (Shin et al., 2022), Tocris], ML-SI 1 [150 µM (Leser et al., 2021), MedChem], NNC 55-0396 [70 µM (Stojanovic et al., 2025), Tocris], ω-agatoxin IVA [200 nM (Beckstead et al., 2004), Alomone Labs], ω-conotoxin GIVA [1 µM (Beckstead et al., 2004), Alomone Labs], paxilline [300 nM (Okhuarobo et al., 2024), Tocris], RY 796 [2.5 µM (Herrington et al., 2011), Alomone Labs], and SNX 482 [100 nM (Siller et al., 2022), Alomone Labs]. Wash-in of quinpirole (30 µM, Sigma-Aldrich) was used to activate the overall pool of D2R in vitro, as described by others (Gantz et al., 2015).

RuBi-dopamine (100 µM, Abcam) was bath applied and uncaged using 100 ms flashes from a 470 nm LED light source (LED4D067 controlled by DC4100, Thorlabs) adjusted to 3 mW.

To selectively stimulate defined presynaptic DA subpopulations, AAV9-based retrograde labeling was used as described previously (Hammer et al., 2024). ChR2-expressing neurons were stimulated with a 470 nm LED (LED4D067, DC4100 controller, Thorlabs) adjusted to 3 mW before each experiment using an optical power meter (PM100, Thorlabs). Three and five stimuli were delivered with a 20 Hz frequency, mimicking mean burst frequencies of 10–20 Hz in vivo (Farassat et al., 2019). For oIPSC recordings, only dʟ-AP5 (10 µM, BioTrend), gabazine (SR95531, 4 µM, Tocris), CNQX (20 µM, Tocris), and CGP55845 (50 nM, Tocris) were applied. Bath application of sulpiride (150 nM, Tocris) abolished the oIPSC. Slices expressing ChR were kept in the dark throughout the whole experiment. In slices where no oIPSC could be elicited, presynaptic neurons were recorded and optically stimulated to check for sufficient ChR expression.

After recordings, slices were fixed overnight at 4°C in 4% paraformaldehyde in phosphate-buffered saline (PBS), pH 7.4, and stored in holding solution (10% sucrose, 0.05% NaN_3_ in H_2_O) at 4°C until immunohistochemistry for post hoc identification.

### Perforated patch-clamp recordings

RB-labeled midbrain DA neurons were recorded using pipettes (GC150TF, Harvard Apparatus) filled with an internal solution containing the following (in mM): 140 KCl, 10 HEPES, 1 EGTA, 2 MgCl_2_, and 0.1% neurobiotin; pH adjusted to 7.35 with KOH, <300 mOsmol. Gramicidin (10–25 µg/ml; 5 mg/ml stock in DMSO) was freshly added before use, as previously described by our group (Lammel et al., 2008). After establishing a gigaseal, membrane permeabilization was monitored until clear action potentials were detected in current-clamp mode.

Slices were continuously superfused with ACSF containing the following(in mM): 125 NaCl, 3.5 KCl, 1.2 MgCl_2_, 1.2 CaCl_2_, 25 NaHCO_3_, and 1.25 NaH_2_PO_4_, bubbled with carbogen (95% O_2_/5% CO_2_). The following blockers were present in all perforated patch experiments: dʟ-AP5 (10 µM, BioTrend), gabazine (4 µM, Tocris), CNQX (20 µM, Tocris), and CGP55845 (50 nM, Tocris). Three minutes after establishing the recording, the above described ACSF additionally containing sulpiride [600 nM (Gantz et al., 2015)] was superfused. After the recording, the cell membrane was ruptured to allow diffusion of neurobiotin for post hoc identification.

### Double patch recordings

For dual-labeled in vitro double patch recordings, DA neurons projecting to the DLS were identified by excitation of red beads, whereas lNAcc-projecting DA neurons were identified by excitation of green beads. Two neurons were recorded simultaneously in current-clamp configuration using pipettes filled with an internal solution containing the following (in mM): 135 K-gluconate, 5 KCl, 10 HEPES, 0.1 EGTA, 5 MgCl_2_, 0.075 CaCl_2_, 5 Na_2_ATP, 1 LiGTP, and 0.1% neurobiotin; pH adjusted to 7.35 with KOH, 290–300 mOsmol. Slices were superfused with ACSF containing the following (in mM): 125 NaCl, 3.5 KCl, 1.2 MgCl_2_, 1.2 CaCl_2_, 25 NaHCO_3_, and 1.25 NaH_2_PO_4_, continuously bubbled with carbogen (95% O_2_/5% CO_2_). The following blockers were present in all double patch experiments: dʟ-AP5 (10 µM, BioTrend), gabazine (SR95531, 4 µM, Tocris), CNQX (20 µM, Tocris), and CGP55845 (50 nM, Tocris). DLS-projecting DA neurons were stimulated by injection of depolarizing current pulses, while spontaneous activity was recorded from lNAcc-projecting neurons (Fig. S3).

For single-labeled in vitro double patch recordings, only lNAcc-projecting neurons were retrogradely labeled with red beads. RB-positive neurons were voltage clamped at −55 mV using an internal solution containing the following (in mM): 115 K-methylsulfonate, 20 NaCl, 1.5 MgCl_2_, 10 BAPTA, 2 Na_2_ATP, 0.2 LiGTP, and 0.1% neurobiotin; pH adjusted to 7.35 with KOH, 290–300 mOsmol, as previously reported to enhance D2R-mediated currents (Gantz et al., 2015). Slices were superfused with ACSF containing the following (in mM): 125 NaCl, 3.5 KCl, 1.2 MgCl_2_, 2.4 CaCl_2_, 25 NaHCO_3_, and 1.25 NaH_2_PO_4_, continuously bubbled with carbogen (95% O_2_/5% CO_2_).

Bead-negative neurons were patched using pipettes filled with an internal solution containing the following (in mM): 135 K-gluconate, 5 KCl, 10 HEPES, 0.1 EGTA, 5 MgCl_2_, 0.075 CaCl_2_, 5 Na_2_ATP, 1 LiGTP, and 0.1% neurobiotin; pH adjusted to 7.35 with KOH, 290–300 mOsmol, and recorded in current-clamp configuration. Negative current was injected to suppress spontaneous pacemaker activity. Positive current pulses (100–300 ms) were applied to evoke 2–4 action potentials every 45 s.

### RABV tracing

cTRIO experiments were performed as previously described (Beier et al., 2015; Beier, 2022), except that TC^66T^ was used rather than TC. For DA_lNAcc_ input tracing, we injected 500 nl of AAV_retro_-DIO-Flp (1.6 × 10^13^ gc/ml) unilaterally into the lNAcc and during the same surgery, also injected 500 nl of a 1:1 volume mix of AAV5-FLEx^FRT^-TC^66T^ (1.3 × 10^13^ gc/ml) and AAV8-FLEx^FRT^-RABV-G (titer 1.3 × 10^12^ gc/ml) into the VTA of DAT-Cre mice. Fourteen days later, a G-deleted, nuc-GFP-expressing, EnvA-pseudotyped RABV (5 × 10^8^ cfu/ml) was injected into the VTA. For DA_DLS_ input tracing, we injected 500 nl AAV_retro_-DIO-Flp (1.6 × 10^13^ gc/ml) unilaterally into the DLS and during the same surgery, also injected 500 nl of a 1:1 volume mix of AAV5-FLEx^FRT^-TC^66T^ (1.3 × 10^13^ gc/ml) and AAV8-FLEx^FRT^-RABV-G (titer 1.3 × 10^12^ gc/ml) into the SN of DAT-Cre mice. Fourteen days later, a G-deleted, nuc-GFP-expressing, EnvA-pseudotyped RABV (5 × 10^8^ cfu/ml) was injected into the SNc. Animals were killed 5 d following RABV injection. Coordinates used for viral injections were as follows (relative to bregma, midline, or dorsal brain surface and in mm): lNAcc: AP +1.45, ML 1.75, DV −4.0; DLS: AP +0.74, ML 2.2, DV −2.6; SN: AP −3.16, ML 1.65, DV −3.8; VTA: AP −3.2, ML 0.4, DV −4.2.

### Immunohistochemistry and confocal microscopy

Coronal sections (60 or100 µm thick) of forebrain tissue containing the striatum or midbrain were prepared using a vibrating microtome (VS1200R, Leica). Striatal and midbrain slices were washed in PBS (0.2 M), pH 7.4, and incubated for 1 h in blocking solution (2 h for midbrain slices) containing 0.2 M PBS supplemented with 10% horse serum, 0.5% Triton X-100, and 0.2% bovine serum albumin (BSA). Slices were then transferred to carrier solution (0.2 M PBS with 1% horse serum, 0.5% Triton X-100, and 0.2% BSA) containing primary antibodies (1:1,000, diluted in carrier solution) and incubated overnight at room temperature. The following primary antibodies were used: monoclonal mouse anti-TH (Merck Millipore, RRID:AB_827536), rabbit anti-TH (EMD Millipore, RRID:AB_390204), polyclonal rabbit anti-TH (Synaptic Systems, RRID:AB_2619896), polyclonal chicken anti-GFP (Abcam, RRID:AB_300798), polyclonal rabbit anti-GFP (Thermo Fisher Scientific, RRID:AB_221569). After 24 h, slices were washed in PBS and incubated overnight at room temperature in carrier solution containing corresponding secondary antibodies (1:750) and Streptavidin conjugates (Alexa Fluor 568, Alexa Fluor 488, or Alexa Fluor 405; 1:1,000, Invitrogen). The following secondary antibodies were used: goat anti-rabbit 568 (Thermo Fisher Scientific, RRID:AB_143157), goat anti-rabbit 488 (Thermo Fisher Scientific, RRID:AB_143165), goat anti-mouse 488 (Thermo Fisher Scientific, RRID:AB_2534069), goat anti-chicken 488 (Abcam, RRID:AB_2827653), donkey anti-rabbit 647 (Molecular Probes, RRID:AB_2536183).

Finally, slices were washed in PBS, mounted in Vectashield (Vector Laboratories), and stored at 4°C. Confocal images were acquired using a laser-scanning microscope (Eclipse 90i, Nikon) controlled by NIS-Elements AR software (Nikon).

### Statistical analysis

Throughout the manuscript, *N* refers to the number of mice, while *n* refers to the number of cells. All analyses were performed using Igor Pro 9 (WaveMetrics) and MATLAB 2025a (MathWorks). Only eIPSCs with peak amplitudes >5.5 pA were included in the analysis of time-to-peak and decay kinetics.

Statistical analyses were carried out using GraphPad Prism 10.6.0 (GraphPad software). Normally distributed data are presented as mean ± SEM, while non-normally distributed data are presented as median with 95% confidence intervals.

*p* values were corrected for multiple comparisons using Bonferroni’s correction.

## Results

### Medial-to-lateral gradient of D2R-eIPSCs is axonal projection specific

To examine potential differences in D2R-mediated eIPSCs among distinct subpopulations of midbrain DA neurons in vitro, we retrogradely labeled DA neurons of adult male C57BL/6N mice by infusing Red Beads (RB) into defined striatal areas (Fig. 1*A*). In acute coronal brain slices, retrogradely labeled DA neurons were visualized, and current responses to bipolar electrical stimulations (three stimuli at 50 Hz; Fig. 1*B*) were recorded, as described previously (Beckstead et al., 2004; Mancini et al., 2025). Under pharmacological isolation, electrical stimulation consistently evoked slow outward currents at a holding potential of −55 mV that were sensitive to the D2R antagonist sulpiride (control: 19.3 pA [17.1–25.8]; sulpiride: 0.0 pA [0.0–0.3]; *n* = 18 cells, *N* = 8 mice; two-tailed Wilcoxon matched-pairs signed rank test, *W* = −171, *p* < 0.0001; Fig. 1*C*,*D*). This confirmed that electrical stimulation evoked DA release that acts upon D2R receptors likely to be coupled to GIRK2 channels [consistent with results from GIRK2-KO mice, where no such currents were observed (Beckstead et al., 2004)]. Red Beads were injected in distinct areas of the striatum—including the lateral shell of nucleus accumbens (lNAcc), the dorsomedial striatum (DMS), the dorsolateral striatum (DLS), or the tail of the striatum (TS)—to retrogradely label midbrain DA neurons according to their axonal projection sites (Fig. 1*E*, top row). In vitro recordings from midbrain slices revealed pronounced differences in D2R-eIPSC amplitudes between these distinct subpopulations of DA neurons. We observed a striking, decreasing medial-to-lateral gradient of eIPSC amplitudes across the midbrain DA neuron subpopulations. DA neurons projecting to the lateral shell of the nucleus accumbens (DA_lNAcc_) exhibited the largest eIPSC amplitudes compared with those projecting to the dorsomedial striatum (DA_DMS_), the dorsolateral striatum (DA_DLS_), and the tail of the striatum (DA_TS_; Fig. 1*E*, bottom row). Thus, DA_lNAcc_ neurons exhibited the largest eIPSC amplitudes with a median of ∼30 pA (DA_lNAcc_: 29.6 pA [24.8–35.4], *n* = 22 cells, *N* = 8 mice; DA_DMS_: 18.5 pA [15.2–21.3], *n* = 22 cells, *N* = 7 mice; DA_DLS_: 12.5 pA [10.6–16.2], *n* = 20 cells, *N* = 7 mice; DA_TS_: 5.4 pA [3.7–9.1], *n* = 12 cells, *N* = 3 mice; one-way ANOVA, *F*_(3,73)_ = 25.74, *p* < 0.0001; Fig. 1*F*). In contrast to different amplitudes, neither the time to peak (DA_lNAcc_: 0.629 ± 0.021 s, *n* = 22 cells, *N* = 8 mice; DA_DMS_: 0.624 ± 0.026 s, *n* = 23 cells, *N* = 7 mice; DA_DLS_: 0.545 ± 0.037 s, *n* = 20 cells, *N* = 7 mice; DA_TS_: 0.675 ± 0.068 s, *n* = 6 cells, *N* = 3 mice; one-way ANOVA, *F*_(3,69)_ = 1.689, *p* = 0.1774; Fig. 1*G*) nor the decay time constant (τ-decay, DA_lNAcc_: 0.821 s [0.778–0.993], *n* = 22 cells, *N* = 8 mice; DA_DMS_: 0.735 s [0.628–0.882], *n* = 23 cells, *N* = 7 mice; DA_DLS_: 0.628 s [0.478–1.062], *n* = 20 cells, *N* = 7 mice; DA_TS_: 0.749 s [0.361–1.102], *n* = 6 cells, *N* = 3 mice; one-way ANOVA, *F*_(3,67)_ = 0.5061, *p* = 0.6794; Fig. 1*H*) differed significantly between DA subpopulations. Mapping of recorded DA neurons along the medio-lateral axis of the midbrain confirmed a projection-specific gradient in eIPSC amplitudes (Fig. 1*I*). Those DA_lNAcc_ neurons located in the lateral ventral tegmental area (lVTA) and medial substantia nigra pars compacta (mSN) exhibited the largest amplitudes. In addition, we extended the dataset by recording DA VTA neurons projecting to the medial shell of nucleus accumbens (DA_mNAcc_; Fig. S1*A–C*). Here, eIPSC amplitudes were smaller (11.2 ± 2.2 pA) than those recorded in DA_lNAcc_ neurons. Furthermore, DA_mNAcc_ neurons exhibited slower eIPSC decays compared with DA_lNAcc_ neurons (Fig. S1*D*,*F*). As described previously, dopamine transporter (DAT) expression differs between DA_lNAcc_ and DA_mNAcc_ neurons (Lammel et al., 2008). Therefore, we tested whether the faster τ-decay observed in DA_lNAcc_ neurons was sensitive to DAT inhibition using the selective inhibitor GBR 12,783 (Fig. S1*E*). As anticipated, DAT inhibition abolished the kinetic decay differences between DA_lNAcc_ neurons and DA_mNAcc_ neurons, indicating lower DAT mediated DA reuptake in DA_mNAcc_ [mNAcc = 1.262 ± 0.165 s, *n* = 12 cells, *N* = 8 mice; lNAcc = 0.821 ± 0.052 s, *n* = 22 cells, *N* = 8 mice; lNAcc_GBR_ = 1.490 ± 0.120 s, *n* = 11 cells, *N* = 3 mice; two-tailed unpaired *t* test (mNAcc vs lNAcc), *t* = 2.701, df = 32, *p* = 0.022; two-tailed unpaired *t* test (mNAcc vs lNAcc_GBR_), *t* = 1.100, df = 21, *p* = 0.5679; Fig. S1*F*].

### Inhibition of presynaptic dendritic but not axonal potassium channels boost eIPSC amplitude in DA_lNAcc_ neurons

**Figure 2.**
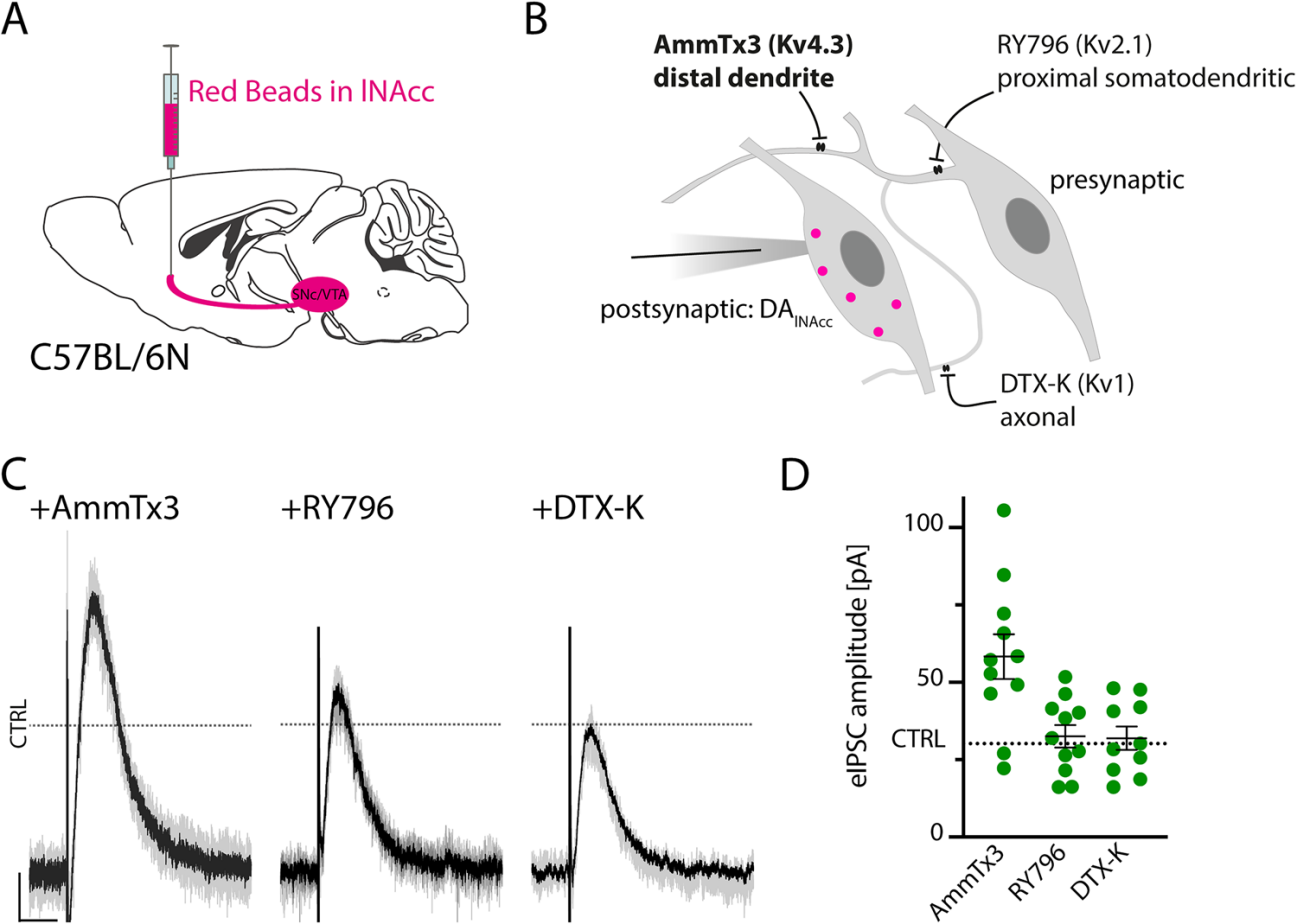
Inhibition of presynaptic dendritic potassium channels boost eIPSC amplitude in DA_lNAcc_ neurons. ***A***, Experimental design. Red Beads were injected into the lateral shell of the nucleus accumbens (lNAcc) of C57BL/6N mice to retrogradely label midbrain DA neurons. ***B***, Schematic illustration of the pharmacological approach used to dissect the contribution of distinct neuronal compartments to DA synaptic release in the midbrain. Selective potassium channel blockers were applied as follows: AmmTx3 to inhibit Kv4.3 channels and probe the distal dendritic compartment; RY 796 to inhibit Kv2.1 channels in proximal dendritic and somatic compartments; and DTX-K to inhibit Kv1.1/Kv1.2 channel-heteromers in axonal compartments. ***C***, Effect of selective potassium-channel blockers on eIPSC amplitude recorded from DA_lNAcc_ neurons. Blocking Kv4.3 channels with AmmTx3 enhanced the eIPSC amplitude, whereas blocking Kv2.1 or Kv1 channels with RY 796 or DTX-K, respectively, did not alter the amplitude. The dashed CTRL line indicates the mean eIPSC amplitude of DA_lNAcc_ neurons under control conditions (at 30.2 pA). Calibration: 10 pA, 1 s. ***D***, Quantitative comparison of eIPSC amplitudes from DA_lNAcc_ neurons following pharmacological inhibition of Kv4, Kv2.1, or Kv1 channels. Blocking Kv4.3 channels with AmmTx3 significantly increased eIPSC amplitude relative to control conditions (eIPSC-DA_lNAcc_), whereas inhibition of Kv2.1 or Kv1 channels had no significant effect. eIPSC-DA_lNAcc_ = 30.2 ± 2.5 pA, *n* = 22 cells, *N* = 4 mice; AmmTx3 = 58.3 ± 7.2 pA, *n* = 11 cells, *N* = 4 mice; RY 796 = 32.6 ± 3.6 pA, *n* = 11 cells, *N* = 3 mice; DTX-K = 31.9 ± 3.8 pA, *n* = 10 cells, *N* = 3 mice; two-tailed unpaired *t* test (eIPSC-DA_lNAcc_ vs AmmTx3), *t* = 4.548, df = 31, *p* < 0.001; two-tailed unpaired *t* test (DA_lNAcc_ vs RY796), *t* = 0.5406, df = 31, *p* = 1; two-tailed unpaired *t* test (DA_lNAcc_ vs DTX-K), *t* = 0.3875, df = 30, *p* = 1; Dashed CTRL line at 30.2 pA. All data are presented as mean ± SEM.

As different potential sites of synaptic contacts between DA neurons in the midbrain have been described using electron microscopy (Wilson et al., 1977; Bayer and Pickel, 1990), we used pharmacological tools to functionally differentiate between different neuronal compartments and their impact on eIPSC amplitude. As we identified DA_lNAcc_ neurons as prominent target for synaptic dopamine transmission within the midbrain, we applied selective potassium channel blockers to probe axonal versus dendritic compartments of the presynaptic DA neurons. We blocked either dendritic Kv4.3 channels (with AmmTx3) to increase the excitability of distal dendritic compartments or Kv2.1 channels (with RY796) to probe somato-dendritic compartments, as well as Kv1.1-Kv1.2 heteromeric channels (with DTX-K) to assess axonal compartments (Fig. 2*A*,*B*). Blocking Kv4.3 channels resulted in a significant twofold increase in eIPSC amplitude in DA_lNAcc_ neurons. In contrast, inhibition of Kv2.1 or Kv1.1/Kv1.2 channels did not affect eIPSC amplitudes in DA_lNAcc_ neurons [eIPSC-DA_lNAcc_ = 30.2 ± 2.5 pA, *n* = 22 cells, *N* = 4 mice; AmmTx3 = 58.3 ± 7.2 pA, *n* = 11 cells, *N* = 4 mice; RY 796 = 32.6 ± 3.6 pA, *n* = 11 cells, *N* = 3 mice; DTX-K = 31.9 ± 3.8 pA, *n* = 10 cells, *N* = 3 mice; two-tailed unpaired *t* test (eIPSC-DA_lNAcc_ vs AmmTx3), *t* = 4.548, df = 31, *p* < 0.001; two-tailed unpaired *t* test (DA_lNAcc_ vs RY796), *t* = 0.5406, df = 31, *p* = 1; two-tailed unpaired *t* test (DA_lNAcc_ vs DTX-K), *t* = 0.3875, df = 30, *p* = 1; Fig. 2*C*,*D*]. Further, inclusion of 4-AP in the internal solution to inhibit postsynaptic Kv4.3 channels did not alter eIPSC amplitudes (Fig. S2*B,C*). These results indicated that DA transmission onto DA_lNAcc_ neurons is boosted by increasing the excitability of the distal dendritic compartment of presynaptic DA neurons.

Notably, inhibition of calcium-activated potassium BK or SK channels did not affect eIPSC amplitude in DA_lNAcc_ neurons (Fig. S2*A–C*). We also examined the contribution of different calcium sources for DA transmission onto DA_lNAcc_ neurons (Fig. S2*D*). Consistent with previous studies (Beckstead et al., 2004; Chen et al., 2006), nonselective inhibition of voltage-gated calcium channels using cadmium completely abolished the eIPSC, whereas D2R-mediated signaling remained intact (Fig. S2*E,F*). In contrast, inhibition of L-type calcium channels increased eIPSC amplitudes, as previously described for SN DA neurons (Gantz et al., 2015), while all other manipulations of distinct calcium sources had no effect on DA_lNAcc_ neurons (Fig. S2*G–J*).

Taken together, our data indicate that the regulation of presynaptic excitability affected the amplitude of postsynaptic eIPSC amplitudes in DA_lNAcc_ neurons.

### Synaptic DA midbrain transmission via optogenetic activation of projection-defined DA subpopulations

**Figure 3.**
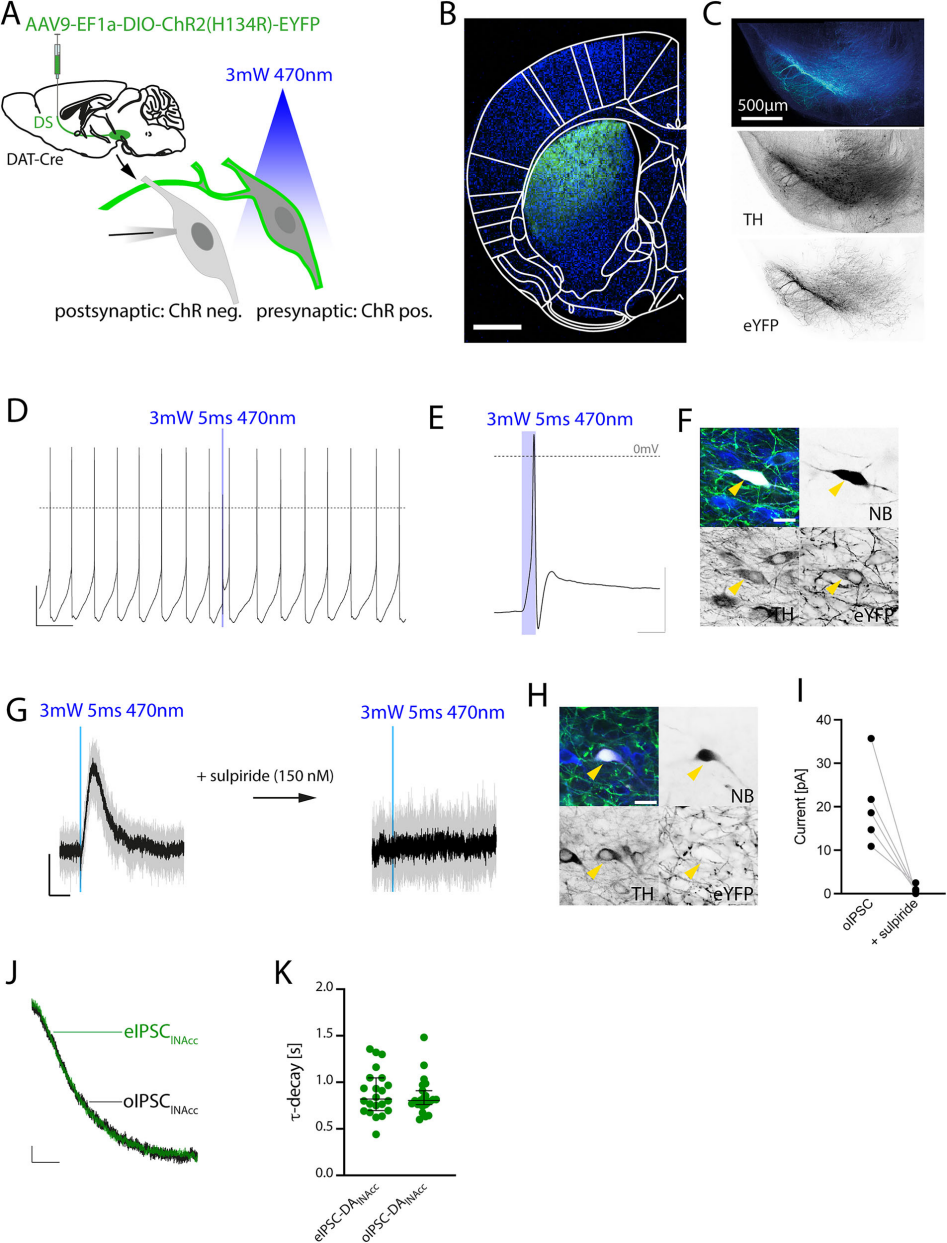
Synaptic DA midbrain transmission via optogenetic activation of projection-defined DA subpopulations. ***A***, Experimental approach for selectively activating midbrain DA neurons defined by their axonal projection site. AAV9-EF1a-DIO-ChR2(H134R)-eYFP was injected into the striatum of DAT-Cre mice to retrogradely infect midbrain DA neurons according to their projection site, enabling optogenetic stimulation of ChR2-positive presynaptic DA neurons with 470 nm light while recording synaptic responses in ChR2-negative neurons. ***B***, eYFP labeling at the striatal injection site confirmed efficient viral expression (blue, DAPI; green, eYFP). Scale bar: 1 mm. ***C***, At the level of the midbrain, eYFP-positive, TH-positive DA neurons were detected in the SN, with dendritic arborizations extending across the SN, VTA, and SNr. Scale bar: 500 µm. ***D***, Wide-field optogenetic stimulation (5 ms pulse, 470 nm, 3 mW) induced additional action potentials in pacemaking midbrain DA neurons. Calibration: 20 mV, 1 s. ***E***, High magnification of the corresponding ChR2-triggered action potential showing characteristic distortions in waveform. ***F***, Post hoc identification of the corresponding recorded DA neuron (yellow arrow) filled with neurobiotin (NB) confirmed eYFP expression and TH positivity in recorded neurons. Calibration: 20 mV, 10 ms. ***G***, Optogenetic stimulation in voltage-clamp mode elicited outward currents (oIPSCs). Bath application of 150 nM sulpiride abolished the outward current, confirming D2R-dependent signaling. Calibration: 10 pA, 1 s. ***H***, Post hoc analysis of the corresponding NB-filled recorded neuron (yellow arrow) confirming the absence of eYFP expression in TH-positive target neurons. ***I***, Quantitative comparison of oIPSC amplitudes before and after sulpiride application in the same cells. Optogenetically evoked oIPSCs were abolished following bath application of 150 nM sulpiride, confirming D2R dependence. oIPSC: 20.3 ± 4.3 pA; sulpiride: 1.0 ± 0.4 pA; *n* = 5 cells, *N* = 4 mice; two-tailed paired *t* test, *t* = 4.225, df = 4, *p* = 0.0134. ***J***, Comparison of normalized decay kinetics of eIPSCs in DA_lNAcc_ neurons (eIPSC_lNAcc_, green trace) with oIPSCs in DA_lNAcc_ after stimulation of DMS-projecting or DLS-projecting DA neurons (oIPSC_lNAcc_, black trace). Calibration: 10%, 1 s. ***K***, τ-decay of oIPSC_lNAcc_ did not differ from eIPSC_lNAcc_. eIPSC_lNAcc_ = 0.885 ± 0.052 s, *n* = 22 cells, *N* = 8 mice; oIPSC_lNAcc_ = 0.913 ± 0.091 s, *n* = 22 cells, *N* = 7 mice; two-tailed Mann–Whitney test, *U* = 224, *p* = 0.6842. All data are presented as mean ± SEM or median (95% CI).

Because the source of presynaptic DA release in the midbrain cannot be resolved using conventional, nonselective electrical stimulation, we employed a projection-specific approach in which defined presynaptic DA neurons were molecularly labeled. Using DAT-Cre mice in combination with stereotactically guided AAV9-mediated injections in striatal subregions, we restricted the expression of channelrhodopsin (ChR)-eYFP to projection-defined subpopulations of DA neurons (e.g., dorsal striatum; Fig. 3*A*).

At the level of the striatum, eYFP-positive terminals were detected at the injection site (Fig. 3*B*), whereas at the level of the midbrain, eYFP- and TH-positive cell bodies were observed (Fig. 3*C*). Optical stimulation of ChR-expressing DA neurons with 5 ms flashes of 470 nm light reliably evoked additional action potentials (Fig. 3*D*). These optogenetically evoked action potentials exhibited a distorted waveform, as described previously (Hammer et al., 2024; Fig. 3*E*). Immunohistochemistry confirmed coexpression of eYFP and TH in the NB-filled recorded neuron (Fig. 3*F*). In a different neuron, the same optical stimulation evoked outward currents that were sensitive to bath application of the D2R antagonist sulpiride (Fig. 3*G*). Immunohistochemistry of the recorded neuron revealed no eYFP expression in the TH-positive, NB-filled midbrain DA neuron (Fig. 3*H*). Across different DA neurons, the amplitudes of the optogenetically evoked outward currents were consistently sensitive to bath application of the D2R antagonist sulpiride (oIPSC: 20.3 ± 4.3 pA; sulpiride: 1.0 ± 0.4 pA; *n* = 5 cells, *N* = 4 mice; two-tailed paired *t* test, *t* = 4.225, df = 4, *p* = 0.0134; Fig. 3*I*). No difference in the decay kinetics between eIPSCs and oIPSCs in DA_lNAcc_ neurons was observed (eIPSC_lNAcc_ = 0.885 ± 0.052 s, *n* = 22 cells, *N* = 8 mice; oIPSC_lNAcc_ = 0.913 ± 0.091 s, *n* = 22 cells, *N* = 7 mice; two-tailed Mann–Whitney test, *U* = 224, *p* = 0.6842; Fig. 3*J*,*K*).

Thus, AAV9-based, ChR-mediated projection-specific stimulation was sufficient to elicit postsynaptic D2R-mediated oIPSCs, demonstrating synaptic DA transmission between distinct midbrain DA neurons.

### Unidirectional, lateral-to-medial synaptic transmission among defined DA subpopulations in the midbrain

**Figure 4.**
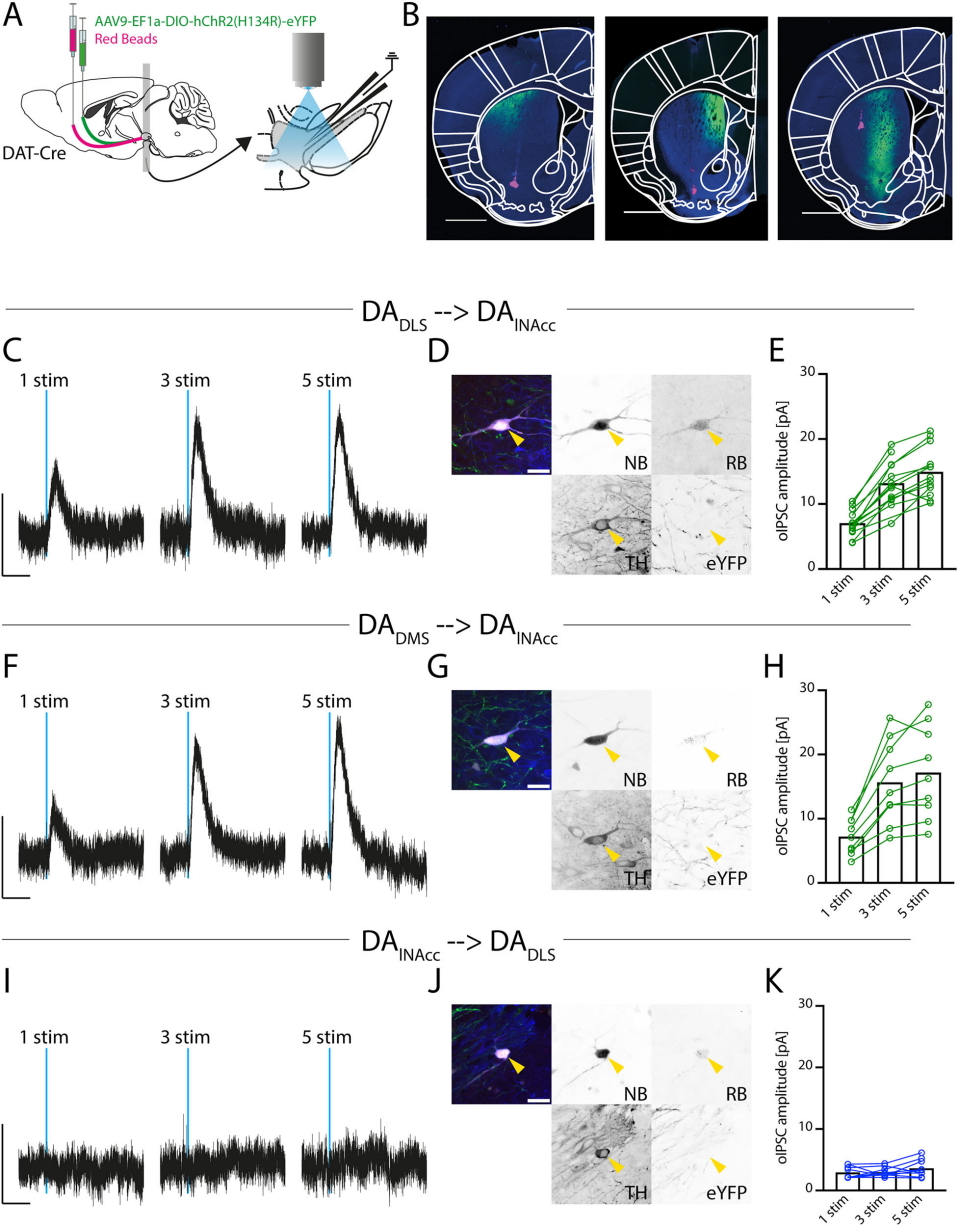
Unidirectional, lateral-to-medial synaptic transmission among defined DA subpopulations in the midbrain. ***A***, Experimental design. DAT-Cre mice received 130 nl injections of AAV9-EF1a-DIO-hChR2(H134R)-eYFP into the DLS, DMS, or lNAcc to retrogradely infect presynaptic midbrain DA neurons. After >28 d, RB were injected into a distinct striatal subregion to retrogradely label potential postsynaptic midbrain DA neurons. Three days later, in vitro patch-clamp recordings were performed. Presynaptic DA neurons were activated by wide-field 470 nm optical stimulation, while postsynaptic DA neurons were recorded in voltage-clamp mode. ***B***, Injection sites for the tested projection combinations. From left to right: AAV9-based retrograde infection of DLS with RB labeling in lNAcc; AAV9-based retrograde infection of DMS with RB labeling in lNAcc; AAV9-based retrograde infection of lNAcc with RB labeling in DLS. Scale bar: 1 mm. ***C***, Optical stimulation of DLS-projecting DA neurons elicited oIPSCs in lNAcc-projecting DA neurons. The oIPSC amplitude approximately doubled with three stimuli compared with one, while further increases to five stimuli produced no additional effect. Calibration: 10 pA, 1 s. ***D***, Post hoc analysis of the recorded DA_lNAcc_ neuron confirmed RB labeling, NB filling, TH positivity, and absence of eYFP expression. ***E***, Mean oIPSC amplitude in DAlNAcc neurons induced by stimulation of DLS-projecting DA neurons. 1 stim = 7.1 ± 0.5 pA, *n* = 14 cells; 3 stim = 13.2 ± 1.0 pA, *n* = 13 cells; 5 stim = 14.9 ± 1.0 pA, *n* = 13 cells, *N* = 4 mice. ***F***, Optical stimulation of DMS-projecting DA neurons also elicited oIPSCs in DA_lNAcc_ neurons. The oIPSC amplitude increased twofold with three stimuli compared with one, with no further enhancement at five stimuli. Calibration: 10 pA, 1 s. ***G***, Post hoc analysis of the recorded DA_lNAcc_ neuron after stimulation of DMS-projecting DA neurons confirmed RB labeling, TH positivity, NB filling, and absence of eYFP expression. ***H***, Mean oIPSC amplitude in DA_lNAcc_ neurons induced by stimulation of DMS-projecting DA neurons. 1 stim = 7.2 ± 0.9 pA, *n* = 9 cells; 3 stim = 15.7 ± 2.2 pA, *n* = 9 cells; 5 stim = 17.2 ± 2.4 pA, *n* = 9 cells, *N* = 3 mice. ***I***, Stimulation of DA_lNAcc_ neurons did not elicit oIPSCs in DLS-projecting DA neurons. Calibration: 10 pA, 1 s. ***J***, Post hoc analysis of the recorded DA_DLS_ neuron confirmed RB labeling, TH positivity, NB filling, and absence of eYFP expression. ***K***, Maximum current within the first second after optical stimulation of lNAcc-projecting DA neurons recorded in DA_DLS_ neurons. 1 stim = 2.9 ± 0.3 pA, *n* = 9 cells; 3 stim = 3.0 ± 0.3 pA, *n* = 9 cells; 5 stim = 3.6 ± 0.5 pA, *n* = 9 cells, *N* = 4 animals. All data are presented as mean ± SEM.

After establishing selective optical stimulation, we examined synaptic DA transmission between defined pre-post subpopulations. We employed a dual-labeling strategy, in which DAT-Cre mice received an injection of AAV9-EF1a-DIO-hChR2(H134R)-eYFP to enable selective optical stimulation of a presynaptic subpopulation of DA neurons. In a second surgery, a postsynaptic DA subpopulation was labeled by retrograde tracing with Red Beads (Fig. 4*A*). We compared three pre-postsynaptic pairs (Fig. 4*B*):

DA_DLS_ → DA_lNAcc_,
DA_DMS_ → DA_lNAcc_
DA_lNAcc_ → DA_DLS_

Selective optical stimulation of DA_DLS_ neurons while recording from DA_lNAcc_ neurons evoked robust oIPSCs (Fig. 4*C–E*, *n* = 14 cells, *N* = 4 mice). Increasing the stimulus number from one to three pulses approximately doubled the oIPSC amplitudes, whereas further increasing the stimulation to five pulses produced little or no additional effect (Fig. 4*C*). This might indicate effective DA release via short bursts of action potentials. Immunohistochemistry confirmed RB labeling and TH expression in the NB-filled recorded neurons, while no eYFP expression was detected (Fig. 4*D*). A stimulus-dependent increase in oIPSC amplitude was consistently observed across recordings (Fig. 4*E*).

In addition, optical stimulation of DA_DMS_ neurons elicited similar oIPSCs in DA_lNAcc_ neurons. Again, the oIPSC amplitudes increased with the number of stimuli (Fig. 4*F*–*H*).

In contrast, reversing the pre-post synaptic order, activation of DA_lNAcc_ neurons did not evoke oIPSCs in DA_DLS_ neurons (*n* = 9 cells, *N* = 3 mice), regardless of the number of stimuli applied (Fig. 4*I*–*K*).

These data demonstrate a unidirectional DA synaptic transmission between distinct subpopulations of DA neurons in the sense that dorsal striatum-projecting, lateral DA neurons provide robust input to ventral striatum-projecting, medial DA neurons but not vice versa.

In an attempt to directly identify individual pre-post synaptic DA pairs, we performed dual whole-cell patch-clamp recordings of projection-identified DA neurons. Despite dual-recordings from >30 pairs of midbrain DA neurons, we found no evidence for DA transmission among them, neither in current-clamp nor voltage-clamp configuration (Figs. S3, S4).

### Rabies tracing confirms sparse, lateral to medial asymmetry of synaptic DA–DA transmission in the midbrain

As a local network between DA neurons in the midbrain has been described anatomically using rabies tracing (Beier, 2022), we employed this approach to test our hypothesis of a unidirectional, asymmetric connectivity between DA_DLS_ and DA_lNAcc_ neurons in the midbrain.

To label local DA inputs onto DA_lNAcc_ neurons, DAT-Cre mice received injections of AAV_retro_-DIO-Flp into the lNAcc and AAV-FLEx^FRT^-TC^66T^-mCherry together with AAV-FLEx^FRT^-RABV-G into the VTA to create and label DA_lNAcc_ as starter cells. Fourteen days later, RABVΔG was injected into the VTA, and 5 d afterward, animals were perfused.

**Figure 5.**
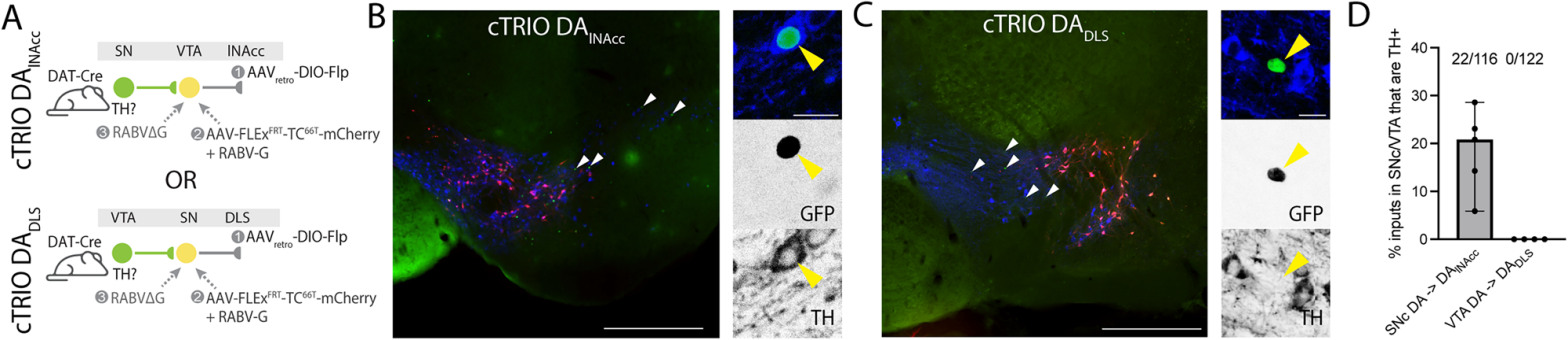
Rabies tracing confirms sparse, lateral to medial asymmetry of synaptic DA–DA transmission in the midbrain. ***A***, Schematic illustration of experimental approach. DAT-Cre mice were injected in two different conditions. To label the local inputs onto DA_lNAcc_ neurons (top row, cTRIO_lNAcc_), AAV_retro_-DIO-Flp was injected in the lNAcc, while a 1:1 mixture of AAV-FLEx^FRT^-TC^66T^-mCherry and AAV-FLEx^FRT^-RABV-G was injected in the VTA. After 14 d, an EnvA-pseudotyped RABVΔG-nucGFP was injected in the VTA to label local inputs onto DA_lNAcc_. To label the local inputs onto DA_DLS_ neurons (bottom row, cTRIO_DLS_), AAV_retro_-DIO-Flp was injected in the DLS, while a 1:1 mixture of AAV-FLEx^FRT^-TC^66T^-mCherry and AAV-FLEx^FRT^-RABV-G was injected in the SN. After 14 d, an EnvA-pseudotyped RABVΔG-nucGFP was injected in the SN to label local inputs onto DA_DLS_. ***B***, cTRIO_lNAcc_ shows labeling in presynaptic neurons in the SN (white arrows), while high magnification images show positive TH and GFP labeling (right column). Scale bars: left, 500 µm; right, 20 µm. ***C***, cTRIO_DLS_ shows labeling in presynaptic neurons in the VTA (white arrows), while high magnification images show no detectable TH immunoreactivity in GFP labeled neurons (right column). Scale bars: left, 500 µm; right, 20 µm. ***D***, Approximately 20% of the inputs to DA_lNAcc_ neurons in the SN were TH-positive (22/116 cells). In contrast, none of the inputs to DA_DLS_ in the VTA were TH-positive (0/122 cells). SNc DA→DA_lNAcc_: 20.8% [5.8–28.6], *N* = 5 mice; VTA DA→DA_DLS_: 0.0% [0.0–0.0], *N* = 4 mice; two-tailed Mann–Whitney test, *U* = 0, *p* = 0.0159. All data are presented as median (95% CI).

To label DA inputs onto DA_DLS_ neurons, DAT-Cre mice received injections of AAV_retro_-DIO-Flp into the DLS and AAV-FLEx^FRT^:TC^66T^-mCherry together with AAV-FLEx^FRT^-RABV-G into the SN. After 14 d, RABVΔG was injected into the SN. Five days later, animals were perfused (Fig. 5*A*). In the cTRIO (cell type-specific tracing the relationship between input and output) DA_lNAcc_ conditions, we detected sparse GFP-positive input from TH-positive DA neurons located in the SN onto DA_lNAcc_ neurons in the VTA (Fig. 5*B*). In contrast, in the cTRIO DA_DLS_ condition, we only detected GFP-positive, TH-negative neurons in the VTA projecting onto DA_DLS_ neurons (Fig. 5*C*). Across all tested animals we detected sparse but reliable TH-positive local input onto DA_lNAcc_ neurons from the SN (∼20%, *N* = 5 mice), while no TH-positive input onto DA_DLS_ neurons was detected in the VTA (*N* = 4 mice; Fig. 5*D*). These data demonstrate an asymmetric cross talk between midbrain DA neurons projecting to distinct target areas of the striatum.

### Medial DA_lNAcc_ neurons possess a fivefold higher synaptic to extrasynaptic D2R ratio compared with lateral DA_DLS_ neurons

**Figure 6.**
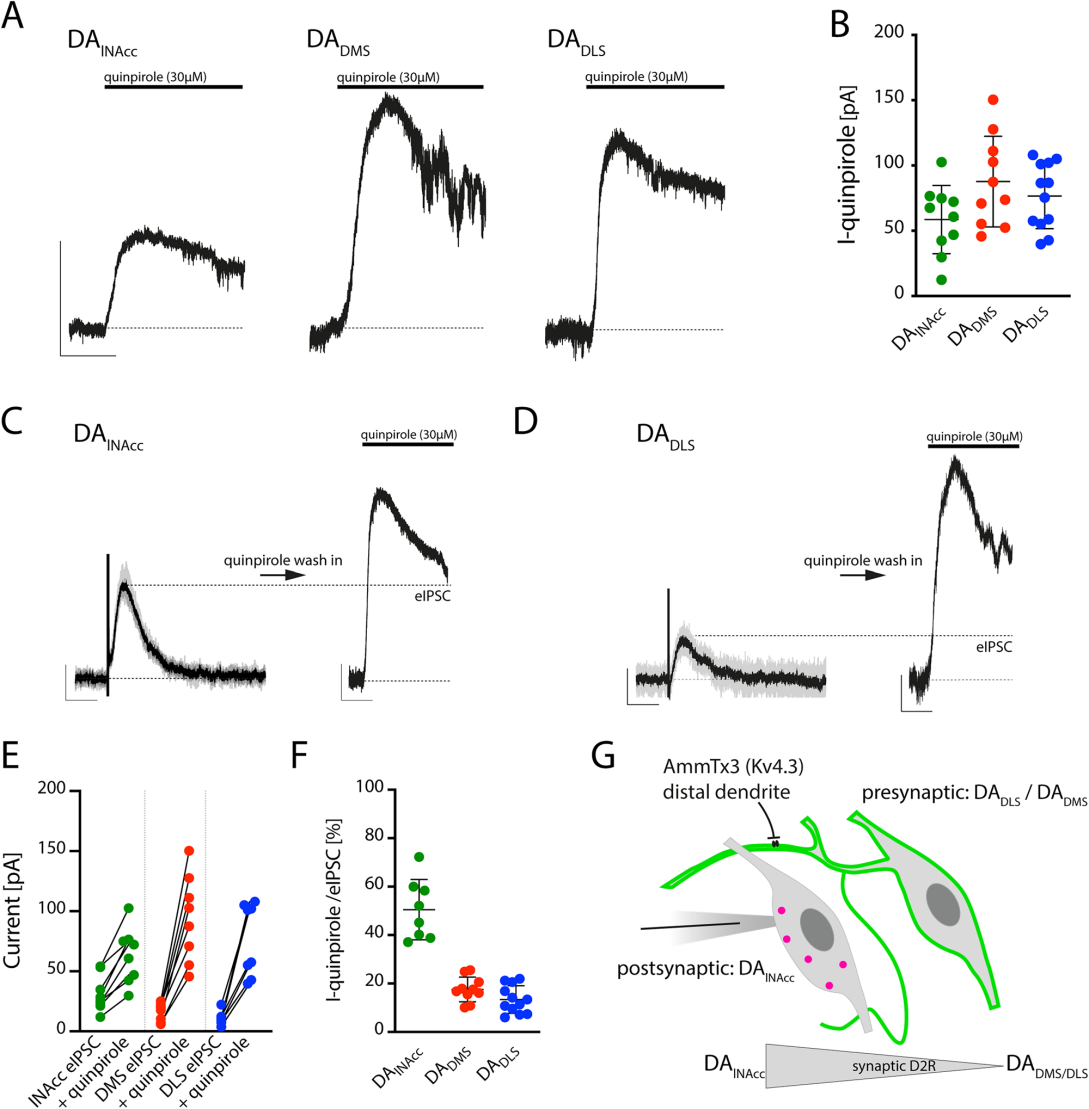
Medial DA_lNAcc_ neurons possess a fivefold higher synaptic to extrasynaptic D2R ratio compared with lateral DA_DLS_ neurons. ***A***, In vitro recordings were performed from retrogradely labeled midbrain DA neurons projecting to the lNAcc, DMS, or DLS from C57BL/6N mice. Neurons were recorded in voltage-clamp configuration at −55 mV. To assess the maximal D2R-mediated current (I-quinpirole), D2Rs were activated by wash-in of a supramaximal concentration of quinpirole (30 µM). Calibration: 50 pA, 1 min. ***B***, A trend toward smaller I-quinpirole amplitudes in lNAcc-projecting DA neurons were observed, compared with I-quinpirole in DMS- and DLS-projecting DA neurons. DA_lNAcc_ = 58.6 ± 8.3 pA, *n* = 10 cells, *N* = 4 mice; DA_DMS_ = 87.7 ± 11.0 pA, *n* = 10 cells, *N* = 4 mice; DA_DLS_ = 76.5 ± 7.2 pA, *n* = 12 cells, *N* = 4 mice; one-way ANOVA, *F*_(2,29)_ = 2.623, *p* = 0.0897. ***C***, Synaptically mediated D2R activation (eIPSCs) was compared with the total D2R pool by recording eIPSCs and I-quinpirole in the same DA_lNAcc_ neuron. Calibration: eIPSC, 20 pA, 1 s; I-quinpirole, 20 pA, 1 min. ***D***, Recordings of eIPSCs and I-quinpirole in the same DA_DLS_ neuron. Calibration: eIPSC, 20 pA, 1 s; I-quinpirole, 20 pA, 1 min. ***E***, Across all recorded DA neurons, I-quinpirole amplitudes were larger than the corresponding eIPSCs, indicating that only a fraction of the total D2R pool is engaged by synaptic DA transmission. eIPSC_lNAcc_ = 31.6 ± 5.5 pA; I-quinpirole_lNAcc_ = 63.6 ± 8.2 pA, *n* = 8 cells, *N* = 4 mice; two-tailed paired *t* test, *t* = 6.829, df = 7, *p* = 0.0002; eIPSC_DMS_ = 16.1 ± 2.6 pA; I-quinpirole_DMS_ = 93.9 ± 12.7 pA, *n* = 8 cells, *N* = 4 mice; two-tailed paired *t* test, *t* = 7.153, df = 7, *p* = 0.0002; eIPSC_DLS_ = 9.4 pA [3.8–22.2]; I-quinpirole_DLS_ = 79.3 pA [39.7–108.0], *n* = 8 cells, *N* = 4 mice; two-tailed Wilcoxon matched-pairs signed rank test, *W* = 36, *p* = 0.0078. ***F***, DA_lNAcc_ neurons exhibited disproportionately high activation of their D2R pool during eIPSC responses compared with DA_DMS_ and DA_DLS_ neurons. Approximately 50% of the available D2R pool was activated through eIPSCs in DA_lNAcc_ neurons, whereas DMS and DLS neurons only utilized <20% of their receptor pool during eIPSCs. DA_lNAcc_ = 50.5 ± 4.4%, *n* = 8 cells, *N* = 4 mice; DA_DMS_ = 17.6 ± 1.6%, *n* = 10 cells, *N* = 4 mice; DA_DLS_ = 13.4 ± 1.6%, *n* = 12 cells, *N* = 4 mice; one-way ANOVA, *F*_(2,27)_ = 59.66, *p* < 0.0001. ***G***, Graphical summary of experimental in vitro findings. DA_lNAcc_ are inhibited through a synapse, where DA is released from DA_DLS_ and DA_DMS_ neurons. In contrast, DA neurons projecting to the dorsal striatum display only limited D2R activation through DA–DA synapses. All data are presented as mean ± SEM or median (95% CI).

In addition to analyzing eIPSC amplitudes, we examined potential differences in the total D2-mediated outward currents by bath application of the D2R agonist quinpirole. Wash-in of supramaximal concentrations of the D2R agonist quinpirole evoked outward currents (I-quinpirole) in DA_lNAcc_, DA_DMS_, and DA_DLS_ neurons (Fig. 6*A*). In contrast to the eIPSC amplitudes, a trend toward larger I-quinpirole amplitudes was observed in DA_DLS_ and DA_DMS_ neurons compared with DA_lNAcc_ neurons (DA_lNAcc_ = 58.6 ± 8.3 pA, *n* = 10 cells, *N* = 4 mice; DA_DMS_ = 87.7 ± 11.0 pA, *n* = 10 cells, *N* = 4 mice; DA_DLS_ = 76.5 ± 7.2 pA, *n* = 12 cells, *N* = 4 mice; one-way ANOVA, *F*_(2,29)_ = 2.623, *p* = 0.0897; Fig. 6*B*). No differences in I-quinpirole desensitization were detected (Fig. S5*A*).

To quantify the fraction of D2Rs activated by synaptic transmission relative to the total D2R pool within a single cell, we combined eIPSC recordings with wash-in of quinpirole in the same neuron (Fig. 6*C*,*D*). All recorded neurons displayed larger I-quinpirole amplitudes compared with their respective eIPSC amplitudes (eIPSC_lNAcc_ = 31.6 ± 5.5 pA; I-quinpirole_lNAcc_ = 63.6 ± 8.2 pA, *n* = 8 cells, *N* = 4 mice; two-tailed paired *t* test, *t* = 6.829, df = 7, *p* = 0.0002; eIPSC_DMS_ = 16.1 ± 2.6 pA; I-quinpirole_DMS_ = 93.9 ± 12.7 pA, *n* = 8 cells, *N* = 4 mice; two-tailed paired *t* test, *t* = 7.153, df = 7, *p* = 0.0002; eIPSC_DLS_ = 9.4 pA [3.8–22.2]; I-quinpirole_DLS_ = 79.3 pA [39.7–108.0], *n* = 8 cells, *N* = 4 mice; two-tailed Wilcoxon matched-pairs signed rank test, W = 36, *p* = 0.0078; Fig. 6*E*). When calculating the ratio of eIPSC amplitude, as a proxy for synaptically activated D2Rs, to the amplitude of I-quinpirole, significant differences between subpopulations were observed. In DA_lNAcc_ neurons, ∼50% of D2Rs were synaptically activated, whereas in dorsostriatal projections only a small fraction (10–20%) was activated through eIPSCs (DA_lNAcc_ = 50.5 ± 4.4%, *n* = 8 cells, *N* = 4 mice; DA_DMS_ = 17.6 ± 1.6%, *n* = 10 cells, *N* = 4 mice; DA_DLS_ = 13.4 ± 1.6%, *n* = 12 cells, *N* = 4 mice; one-way ANOVA, *F*_(2,27)_ = 59.66, *p* < 0.0001; Fig. 6*F*).

Despite the distinct ratios of synaptic to extrasynaptic of D2R activation across the tested subpopulations, all groups were tonically inhibited by ongoing D2R activation in vitro, indicating a high sensitivity of their pacemaker activity to tonic D2R signaling (Fig. S6).

Hence, in lNAcc-projecting DA neurons somato-dendritic D2Rs might be activated to a higher extend by synaptically released DA, in contrast to somato-dendritic D2Rs in DMS and DLS-projecting DA neurons.

Overall, our in vitro experiments indicated a unidirectional synaptic DA transmission between defined subpopulations of midbrain DA neurons. On the one hand, in DA_lNAcc_ neurons half of their D2R pool is activated through synaptic DA transmission with presynaptic DA partners projecting to dorsal striatum. On the other hand, DA_DLS_ neurons receive little to no synaptic input from other DA neurons, despite a similar overall D2R-pool, possibly sensing, e.g., DA volume transmission or autoinhibition (Fig. 6*G*).

## Discussion

Our study identified the pre- and postsynaptic partners of dopamine synapses in the midbrain, both by functional recordings as well as by rabies virus monosynaptic tracing. This DA synapse is predominantly constituted in a motor-to-limbic direction by presynaptic nigrostriatal DA neurons (projecting to the dorsal striatum) and postsynaptic mesolimbic DA neurons [projecting to the lateral shell of the nucleus accumbens (DA_lNAcc_)]. In contrast, reverse, limbic-to-motor DA synapses were not observed. Thus, the DA synapse establishes a unidirectional communication from nigrostriatal DA onto mesolimbic DA neurons. The sum of the average oIPSC amplitudes in DA_lNAcc_ neuron from DLS- and DMS-projecting DA neurons was very similar to the evoked eIPSC in DA_lNAcc_ neurons. This might indicate that DLS-projecting and DMS-projecting DA neurons converge with their DA synapses onto mesolimbic DA neurons. To fully activate the pool of DA synapses present in the slice, we used stimulus trains of three to five pulses, consistent with the number of action potentials per burst observed in dorsal striatum-projecting midbrain DA neurons in vivo under anesthesia (Farassat et al., 2019). This facilitation pattern aligns with previous reports demonstrating enhanced dopamine release during trains of stimuli (Beckstead et al., 2007). The amplitude range of oIPSCs and previously reported minis (Gantz et al., 2013) suggests a presynaptic pool of ∼10 nigrostriatal DA synapses onto mesolimbic DA neuron.

### Properties of DA synaptic transmission onto DA_lNAcc_

The biophysical properties of the DA–DA synapse in this study were very similar to previous foundational work by the Williams laboratory (Beckstead et al., 2004). Our selective inhibition of axonal and dendritically localized K^+^ channels provided evidence that only increased dendritic excitability by inhibition of Kv4.3 channels in presynaptic DA neurons enhanced the amplitude of postsynaptic eIPSCs. The results are consistent with both increased dendritic or axonal DA release given that increased dendritic excitability might enhance axon-dendritic coupling. Therefore, these pharmacological experiments did not define the dendritic or axonal localization of the DA synapse.

### Extrasynaptic DA signaling

In contrast to DA_lNAcc_ neurons, DA neurons projecting to the DS recruited only ∼20% of their D2Rs through eIPSCs. This is consistent with anatomical data where only ∼7% of D2Rs in SN DA neurons were located within dendro-dendritic synapses (Lebowitz et al., 2022). These ratio might not be absolute but are likely to be affected by incomplete activation of synaptic D2R through our stimulation protocol (Beckstead et al., 2007) and Ca^2+^-dependent desensitization of D2R (Gantz et al., 2015). Given these caveats, it is still likely that a significant component of DA signaling in the midbrain—particular in nigrostriatal neurons—is extrasynaptic (Wilson et al., 1977; Lebowitz et al., 2022).

### Limitations of the study

Our in vitro study was carried out in 250 µm coronal slices of adult mice. Given the morphological reconstructions of DA_lNAcc_ (Montero et al., 2021; Hammer et al., 2024; Gatica et al., 2025), DA axons and some distal DA dendrites are expected to have been cut, and consequently, distal DA synapses might have been lost. Thus, our functional characterization of DA–DA synapses is likely to be incomplete. However, this limitation does not apply to our complementary monosynaptic rabies approach carried out in vivo. Also, we have not attempted to identify D2R-mediated cross talk within single DA projections that expressed channelrhodopsin for selective stimulation. Finally, our study did not resolve the localization, axo-dendritic and/or dendro-dendritic of the motor-to-limbic DA synapse.

### Hypothesis for functional relevance of a motor-to-limbic DA synapse

We showed that DA_lNAcc_ neurons are a main target of synaptic DA signaling in the midbrain, receiving direct inhibitory input from nigrostriatal DA neurons. Therefore, this DA synapse might serve for a direct inhibitory communication between senso-motor and mesolimbic basal ganglia circuits. This communication is in the opposite direction to the ascending, polysynaptic, limbic-to-motor spiral in primates (Haber et al., 2000; Haber, 2003). Recent studies in rodents described prominent closed-loop basal ganglia circuits in parallel (Ambrosi and Lerner, 2022).

We speculate that nigrostriatal DA neurons, which increase their activity upon the onset of motor activity (da Silva et al., 2018), might inhibit DA_lNAcc_ neurons during the execution of movements, thus mediating a motor-to-limbic DA inhibition. We assume that this inhibition would be most efficient during burst activity in the presynaptic DA neurons. This pattern has recently been described in vivo for lateral VTA DA neurons after the onset of licking (de Jong et al., 2024). However, future in vivo studies combining recording of projection-defined DA neurons with a cell-selective D2R manipulations [e.g., by DART systems (Shields et al., 2024) or opto-switchable D2R (Donthamsetti et al., 2017)] are needed to probe functional ideas about the motor-to-limbic DA synapse.

## References

[B1] Ambrosi P, Lerner TN (2022) Striatonigrostriatal circuit architecture for disinhibition of dopamine signaling. Cell Rep 40:111228. 10.1016/j.celrep.2022.111228 35977498 PMC9425427

[B2] Bayer VE, Pickel VM (1990) Ultrastructural localization of tyrosine hydroxylase in the rat ventral tegmental area: relationship between immunolabeling density and neuronal associations. J Neurosci 10:2996–3013. 10.1523/JNEUROSCI.10-09-02996.1990 1975839 PMC6570237

[B3] Beckstead MJ, Grandy DK, Wickman K, Williams JT (2004) Vesicular dopamine release elicits an inhibitory postsynaptic current in midbrain dopamine neurons. Neuron 42:939–946. 10.1016/j.neuron.2004.05.019 15207238

[B4] Beckstead MJ, Ford CP, Phillips PE, Williams JT (2007) Presynaptic regulation of dendrodendritic dopamine transmission. Eur J Neurosci 26:1479–1488. 10.1111/j.1460-9568.2007.05775.x 17822435 PMC3633601

[B5] Beier K (2022) Modified viral-genetic mapping reveals local and global connectivity relationships of ventral tegmental area dopamine cells. eLife 11:e76886. 10.7554/eLife.76886 35604019 PMC9173742

[B6] Beier KT, Steinberg EE, DeLoach KE, Xie S, Miyamichi K, Schwarz L, Gao XJ, Kremer EJ, Malenka RC, Luo L (2015) Circuit architecture of VTA dopamine neurons revealed by systematic input-output mapping. Cell 162:622–634. 10.1016/j.cell.2015.07.015 26232228 PMC4522312

[B7] Chen BT, Moran KA, Avshalumov MV, Rice ME (2006) Limited regulation of somatodendritic dopamine release by voltage-sensitive Ca channels contrasted with strong regulation of axonal dopamine release. J Neurochem 96:645–655. 10.1111/j.1471-4159.2005.03519.x 16405515

[B8] Chen BT, Patel JC, Moran KA, Rice ME (2011) Differential calcium dependence of axonal versus somatodendritic dopamine release, with characteristics of both in the ventral tegmental area. Front Syst Neurosci 5:39. 10.3389/fnsys.2011.00039 21716634 PMC3115476

[B9] Coddington LT, Dudman JT (2019) Learning from action: reconsidering movement signaling in midbrain dopamine neuron activity. Neuron 104:63–77. 10.1016/j.neuron.2019.08.036 31600516

[B10] da Silva JA, Tecuapetla F, Paixão V, Costa RM (2018) Dopamine neuron activity before action initiation gates and invigorates future movements. Nature 554:244–248. 10.1038/nature25457 29420469

[B11] de Jong JW, Afjei SA, Pollak Dorocic I, Peck JR, Liu C, Kim CK, Tian L, Deisseroth K, Lammel S (2019) A neural circuit mechanism for encoding aversive stimuli in the mesolimbic dopamine system. Neuron 101:133–151 e137. 10.1016/j.neuron.2018.11.005 30503173 PMC6317997

[B12] de Jong JW, Liang Y, Verharen JPH, Fraser KM, Lammel S (2024) State and rate-of-change encoding in parallel mesoaccumbal dopamine pathways. Nat Neurosci 27:309–318. 10.1038/s41593-023-01547-6 38212586 PMC11590751

[B13] Donthamsetti PC, Winter N, Schonberger M, Levitz J, Stanley C, Javitch JA, Isacoff EY, Trauner D (2017) Optical control of dopamine receptors using a photoswitchable tethered inverse agonist. J Am Chem Soc 139:18522–18535. 10.1021/jacs.7b07659 29166564 PMC5942546

[B14] Farassat N, et al. (2019) In vivo functional diversity of midbrain dopamine neurons within identified axonal projections. eLife 8:e48408. 10.7554/eLife.48408 31580257 PMC6791716

[B15] Ferraro L, Frankowska M, Marcellino D, Zaniewska M, Beggiato S, Filip M, Tomasini MC, Antonelli T, Tanganelli S, Fuxe K (2012) A novel mechanism of cocaine to enhance dopamine d2-like receptor mediated neurochemical and behavioral effects. An in vivo and in vitro study. Neuropsychopharmacology 37:1856–1866. 10.1038/npp.2012.33 22453136 PMC3376318

[B17] Ford CP (2014) The role of D2-autoreceptors in regulating dopamine neuron activity and transmission. Neuroscience 282:13–22. 10.1016/j.neuroscience.2014.01.025 24463000 PMC4108583

[B16] Ford CP, Phillips PE, Williams JT (2009) The time course of dopamine transmission in the ventral tegmental area. J Neurosci 29:13344–13352. 10.1523/JNEUROSCI.3546-09.2009 19846722 PMC2791792

[B18] Gantz SC, Bunzow JR, Williams JT (2013) Spontaneous inhibitory synaptic currents mediated by a G protein-coupled receptor. Neuron 78:807–812. 10.1016/j.neuron.2013.04.013 23764286 PMC3697754

[B19] Gantz SC, Robinson BG, Buck DC, Bunzow JR, Neve RL, Williams JT, Neve KA (2015) Distinct regulation of dopamine D2S and D2L autoreceptor signaling by calcium. eLife 4:e09358. 10.7554/eLife.09358 26308580 PMC4575989

[B20] Gantz SC, Ford CP, Morikawa H, Williams JT (2018) The evolving understanding of dopamine neurons in the substantia nigra and ventral tegmental area. Annu Rev Physiol 80:219–241. 10.1146/annurev-physiol-021317-121615 28938084

[B21] Gatica RI, Montero T, Farassat N, Henny P (2025) Geometrical factors determining dendritic domain intersection between neurons: a modeling study. Brain Struct Funct 230:154. 10.1007/s00429-025-03011-6 41081905

[B22] Geffen LB, Jessell TM, Cuello AC, Iversen LL (1976) Release of dopamine from dendrites in rat substantia nigra. Nature 260:258–260. 10.1038/260258a0 1256567

[B23] Gittelman JX, Tempel BL (2006) Kv1.1-containing channels are critical for temporal precision during spike initiation. J Neurophysiol 96:1203–1214. 10.1152/jn.00092.2005 16672305

[B24] Grace AA (2016) Dysregulation of the dopamine system in the pathophysiology of schizophrenia and depression. Nat Rev Neurosci 17:524–532. 10.1038/nrn.2016.57 27256556 PMC5166560

[B25] Groves PM, Linder JC (1983) Dendro-dendritic synapses in substantia-nigra - descriptions based on analysis of serial sections. Exp Brain Res 49:209–217. 10.1007/BF00238581 6832258

[B27] Haber SN (2003) The primate basal ganglia: parallel and integrative networks. J Chem Neuroanat 26:317–330. 10.1016/j.jchemneu.2003.10.003 14729134

[B26] Haber SN, Fudge JL, McFarland NR (2000) Striatonigrostriatal pathways in primates form an ascending spiral from the shell to the dorsolateral striatum. J Neurosci 20:2369–2382. 10.1523/JNEUROSCI.20-06-02369.2000 10704511 PMC6772499

[B28] Hammer N, Vogel P, Lee S, Roeper J (2024) Optogenetic action potentials and intrinsic pacemaker interplay in retrogradely identified midbrain dopamine neurons. Eur J Neurosci 59:1311–1331. 10.1111/ejn.16208 38056070

[B29] Herrington J, et al. (2011) Identification of novel and selective Kv2 channel inhibitors. Mol Pharmacol 80:959–964. 10.1124/mol.111.074831 21948463

[B30] Hikima T, Lee CR, Witkovsky P, Chesler J, Ichtchenko K, Rice ME (2021) Activity-dependent somatodendritic dopamine release in the substantia nigra autoinhibits the releasing neuron. Cell Rep 35:108951. 10.1016/j.celrep.2021.108951 33826884 PMC8189326

[B31] Lacey MG, Mercuri NB, North RA (1987) Dopamine acts on D2 receptors to increase potassium conductance in neurones of the rat substantia nigra zona compacta. J Physiol 392:397–416. 10.1113/jphysiol.1987.sp016787 2451725 PMC1192311

[B32] Lammel S, Hetzel A, Hackel O, Jones I, Liss B, Roeper J (2008) Unique properties of mesoprefrontal neurons within a dual mesocorticolimbic dopamine system. Neuron 57:760–773. 10.1016/j.neuron.2008.01.022 18341995

[B33] Lebowitz JJ, Trinkle M, Bunzow JR, Balcita-Pedicino JJ, Hetelekides S, Robinson B, De La Torre S, Aicher SA, Sesack SR, Williams JT (2022) Subcellular localization of D2 receptors in the murine substantia nigra. Brain Struct Funct 227:925 941. 10.1007/s00429-021-02432-3 34854963 PMC8930450

[B34] Ledonne A, Mercuri NB (2018) mGluR1-dependent long term depression in rodent midbrain dopamine neurons is regulated by neuregulin 1/ErbB signaling. Front Mol Neurosci 11:346. 10.3389/fnmol.2018.00346 30327588 PMC6174199

[B35] Lerner TN, et al. (2015) Intact-brain analyses reveal distinct information carried by SNc dopamine subcircuits. Cell 162:635–647. 10.1016/j.cell.2015.07.014 26232229 PMC4790813

[B36] Leser C, Keller M, Gerndt S, Urban N, Chen CC, Schaefer M, Grimm C, Bracher F (2021) Chemical and pharmacological characterization of the TRPML calcium channel blockers ML-SI1 and ML-SI3. Eur J Med Chem 210:112966. 10.1016/j.ejmech.2020.112966 33187805

[B37] Liss B, Haeckel O, Wildmann J, Miki T, Seino S, Roeper J (2005) K-ATP channels promote the differential degeneration of dopaminergic midbrain neurons. Nat Neurosci 8:1742–1751. 10.1038/nn1570 16299504

[B38] Mancini M, Hikima T, Witkovsky P, Patel JC, Stone DW, Affinati AH, Rice ME (2025) Leptin activates dopamine and GABA neurons in the substantia nigra via a local pars compacta-pars reticulata circuit. J Neurosci 45:e1539242025. 10.1523/JNEUROSCI.1539-24.2025 40127936 PMC12096038

[B39] Markowitz JE, et al. (2023) Spontaneous behaviour is structured by reinforcement without explicit reward. Nature 614:108–117. 10.1038/s41586-022-05611-2 36653449 PMC9892006

[B40] McCall NM, Kotecki L, Dominguez-Lopez S, Marron Fernandez de Velasco E, Carlblom N, Sharpe AL, Beckstead MJ, Wickman K (2017) Selective ablation of GIRK channels in dopamine neurons alters behavioral effects of cocaine in mice. Neuropsychopharmacology 42:707–715. 10.1038/npp.2016.138 27468917 PMC5240170

[B41] Menegas W, Babayan BM, Uchida N, Watabe-Uchida M (2017) Opposite initialization to novel cues in dopamine signaling in ventral and posterior striatum in mice. eLife 6:e10032. 10.7554/eLife.10032 PMC5271609 28054919

[B42] Montero T, Gatica RI, Farassat N, Meza R, Gonzalez-Cabrera C, Roeper J, Henny P (2021) Dendritic architecture predicts in vivo firing pattern in mouse ventral tegmental area and substantia nigra dopaminergic neurons. Front Neural Circuits 15:769342. 10.3389/fncir.2021.769342 34867214 PMC8640462

[B43] Morales M, Margolis EB (2017) Ventral tegmental area: cellular heterogeneity, connectivity and behaviour. Nat Rev Neurosci 18:73–85. 10.1038/nrn.2016.165 28053327

[B44] Okhuarobo A, et al. (2024) Ethanol’s interaction with BK channel alpha subunit residue K361 does not mediate behavioral responses to alcohol in mice. Mol Psychiatry 29:529–542. 10.1038/s41380-023-02346-y 38135755 PMC11116116

[B45] Poulin JF, Caronia G, Hofer C, Cui Q, Helm B, Ramakrishnan C, Chan CS, Dombeck DA, Deisseroth K, Awatramani R (2018) Mapping projections of molecularly defined dopamine neuron subtypes using intersectional genetic approaches. Nat Neurosci 21:1260–1271. 10.1038/s41593-018-0203-4 30104732 PMC6342021

[B46] Schultz W, Dayan P, Montague PR (1997) A neural substrate of prediction and reward. Science 275:1593–1599. 10.1126/science.275.5306.1593 9054347

[B47] Sesack SR, Aoki C, Pickel VM (1994) Ultrastructural localization of D2 receptor-like immunoreactivity in midbrain dopamine neurons and their striatal targets. J Neurosci 14:88–106. 10.1523/JNEUROSCI.14-01-00088.1994 7904306 PMC6576858

[B48] Shields BC, et al. (2024) DART.2: bidirectional synaptic pharmacology with thousandfold cellular specificity. Nat Methods 21:1288–1297. 10.1038/s41592-024-02292-9 38877316 PMC11569460

[B49] Shin J, et al. (2022) Ca(v)1.3 calcium channels are full-range linear amplifiers of firing frequencies in lateral DA SN neurons. Sci Adv 8:eabm4560. 10.1126/sciadv.abm4560 35675413 PMC9177074

[B50] Siller A, et al. (2022) beta2-subunit alternative splicing stabilizes Cav2.3 Ca(2+) channel activity during continuous midbrain dopamine neuron-like activity. eLife 11:e67464. 10.7554/eLife.67464 35792082 PMC9307272

[B51] Stojanovic S, Knowlton CJ, Egger-Mackrodt R, Mankel J, Shin J, Lammel S, Canavier CC, Roeper J (2025) Rebound bursting selectively enables fast dynamics in dopamine midbrain neurons projecting to the dorso-lateral striatum. J Neurosci 45:e0361252025. 10.1523/JNEUROSCI.0361-25.2025 41006064 PMC12572932

[B52] Surmeier DJ, Obeso JA, Halliday GM (2017) Selective neuronal vulnerability in Parkinson disease. Nat Rev Neurosci 18:101–113. 10.1038/nrn.2016.178 28104909 PMC5564322

[B53] Tsuneki H, Klink R, Léna C, Korn H, Changeux JP (2000) Calcium mobilization elicited by two types of nicotinic acetylcholine receptors in mouse substantia nigra pars compacta. Eur J Neurosci 12:2475–2485. 10.1046/j.1460-9568.2000.00138.x 10947823

[B54] Volkow ND, Wise RA, Baler R (2017) The dopamine motive system: implications for drug and food addiction. Nat Rev Neurosci 18:741–752. 10.1038/nrn.2017.130 29142296

[B55] Watabe-Uchida M, Zhu L, Ogawa SK, Vamanrao A, Uchida N (2012) Whole-brain mapping of direct inputs to midbrain dopamine neurons. Neuron 74:858–873. 10.1016/j.neuron.2012.03.017 22681690

[B56] Wilson CJ, Groves PM, Fifkova E (1977) Monoaminergic synapses, including dendro-dendritic synapses in the rat substantia nigra. Exp Brain Res 30:161–174. 10.1007/BF00237248 598426

[B57] Zhuang X, Masson J, Gingrich JA, Rayport S, Hen R (2005) Targeted gene expression in dopamine and serotonin neurons of the mouse brain. J Neurosci Methods 143:27–32. 10.1016/j.jneumeth.2004.09.020 15763133

